# Control of Immediate Early Gene Expression for Human Cytomegalovirus Reactivation

**DOI:** 10.3389/fcimb.2020.00476

**Published:** 2020-09-17

**Authors:** Donna Collins-McMillen, Jeremy Kamil, Nathaniel Moorman, Felicia Goodrum

**Affiliations:** ^1^Department of Immunobiology and BIO5 Institute, University of Arizona, Tucson, AZ, United States; ^2^Department of Microbiology and Immunology, Louisiana State University Health Sciences Center - Shreveport, Shreveport, LA, United States; ^3^Department of Microbiology and Immunology, Lineberger Comprehensive Cancer Center, University of North Carolina at Chapel Hill, Chapel Hill, NC, United States

**Keywords:** cytomegalovirus, alternative promoter usage, herpesvirus, latency, reactivation, major immediate early (MIE) promoter

## Abstract

Human cytomegalovirus (HCMV) is a beta herpesvirus that persists for life in the majority of the world's population. The persistence of HCMV in the human population is due to the exquisite ability of herpesviruses to establish a latent infection that evades elimination by the host immune response. How the virus moves into and out of the latent state has been an intense area of research focus and debate. The prevailing paradigm is that the major immediate early promoter (MIEP), which drives robust expression of the major immediate early (MIE) transactivators, is epigenetically silenced during the establishment of latency, and must be reactivated for the virus to exit latency and re-enter productive replication. While it is clear that the MIEP is silenced by the association of repressive chromatin remodeling factors and histone marks, the mechanisms by which HCMV de-represses MIE gene expression for reactivation are less well understood. We have identified alternative promoter elements within the MIE locus that drive a second or delayed phase of MIE gene expression during productive infection. In the context of reactivation in THP-1 macrophages and primary CD34+ human progenitor cells, MIE transcripts are predominantly derived from initiation at these alternative promoters. Here we review the mechanisms by which alternative viral promoters might tailor the control of viral gene expression and the corresponding pattern of infection to specific cell types. Alternative promoter control of the HCMV MIE locus increases versatility in the system and allows the virus to tightly repress viral gene expression for latency but retain the ability to sense and respond to cell type-specific host cues for reactivation of replication.

Human cytomegalovirus (HCMV) is one of nine herpesviruses infecting humans. Primary HCMV infection is typically asymptomatic. Like all herpesviruses, HCMV evades immune clearance and establishes a life-long latent persistence (Goodrum, 2016). The latent persistence is marked by sporadic but likely frequent and typically subclinical reactivation events. Reactivation results in serious disease risk in those undergoing immune suppressive therapy associated with solid organ or stem cell transplantation or intensive chemotherapy associated with cancer treatment. In healthy adults, the cost or benefit of asymptomatic HCMV carriage is not well understood. HCMV seropositivity is associated with increased risk for cardiovascular disease and immune dysfunction in the aged (Streblow et al., 2008; Nikolich-Zugich et al., 2017). However, there is also evidence that HCMV persistence may boost immune responses to infection or vaccination (Furman et al., 2015).

HCMV infects a diverse array of human cell types. While the biology of HCMV infection has been most thoroughly characterized in the context of robust productive replication in fibroblasts, other patterns of infection are unique to other cell types. For example, replication in epithelial cells and endothelial cells results in a chronic or low-level smoldering infection (Jarvis and Nelson, 2002, 2007; Adler and Sinzger, 2009). The factors driving these different infection outcomes are currently unclear, as the differences in viral gene expression or the manipulation of host pathways associated with infection in these cell types have not been systematically dissected relative to infection in fibroblasts. The latent pattern of HCMV infection is thought to be restricted to hematopoietic progenitors or cells of the myeloid lineage, and has been most thoroughly characterized in CD34+ hematopoietic progenitors (HPCs) (Goodrum, 2016). The ability of HCMV to infect and establish unique patterns of infection in a variety of cell types is indicative of the exceptional complexity the virus has attained through its co-evolution with its host, and is undoubtedly key to its lifelong persistence. Cell type-specific regulation of viral patterns of infection is a significant gap in our understanding of HCMV persistence. In this review, we focus on the regulation of re-expression of major immediate early genes following reactivation.

During latency, the viral genome is maintained in the absence of virus replication (Goodrum, 2016). Viral gene expression associated with latency has been challenging to define due to the heterogeneity of hematopoietic cell subpopulations. Conventionally, herpesvirus latency has been thought to be a silent state where viral gene expression is restricted to a limited number of genes—the so-called latency genes. However, recent transcriptomics studies across the herpesvirus field, suggest a much more active and dynamic state of latency (Collins-McMillen and Goodrum, 2017; Singh and Tscharke, 2020). In HCMV infection of hematopoietic cells, the viral genes are broadly expressed, but at exceptionally low levels (Cheng et al., 2017; Shnayder et al., 2018). These findings have complicated defining distinct latent patterns of gene expression or the use of IE gene expression alone as an indicator of reactivation. Nevertheless, many studies have used the relative levels of immediate early or replicative genes (e.g., IE-86kDa, IE2-72kDa, UL135) to latency genes (e.g., UL138, LUNA) to define the latent state where the ratio of replicative to latent genes is greater in replicative states than in latent states (Kim et al., 2017; Shnayder et al., 2018; Buehler et al., 2019; Krishna et al., 2019).

In latency, viral gene expression is restricted by chromatinization and epigenetic regulation of the viral chromosome (Murphy et al., 2002; Reeves et al., 2005; Abraham and Kulesza, 2013; Albright and Kalejta, 2016). Studies of chromatin changes associated with latency and reactivation in HCMV have primarily focused on the major immediate early promoter (MIEP) (Woodhall et al., 2006; Groves et al., 2009). The MIEP is a complex promoter, consisting of an enhancer (−520 to −65 nucleotides, nt), a unique region (−780 to −610 nt), and a modulator (−1145 to −750 nt) in addition to the core promoter (−65 to +3 nt). While the promoter is sufficient for transcription of IE genes (Thomsen et al., 1984), the enhancer element strongly enhances transcription (Boshart et al., 1985). The modulator has context dependent roles, repressive to MIEP activity in undifferentiated cells, but actively drives the MIEP in permissive fibroblasts (Nelson et al., 1987; Lubon et al., 1989; Shelbourn et al., 1989; Huang et al., 1996). The MIEP drives robust expression of the major immediate early genes, UL122 and UL123, encoding a number of major immediate early proteins, most notably the IE1-72kDa and IE2-86kDa major transactivators. The MIEP is initially transactivated by host transcription factors and gives rise to a common RNA precursor that is alternatively spliced (Stenberg et al., 1985; Liu and Stinski, 1992; Collins-McMillen et al., 2018). IE1-72kDa is encoded by exons 1-4 and IE2-86kDa is produced by an exon skipping event and is encoded by exons 1-3 and 5. Preferential splicing results in early accumulation of IE1, which switches at later times to favor IE2 (Sanchez et al., 2004; Oduro et al., 2012; Lin et al., 2017).

Chromatinization and silencing of the viral genome has been broadly studied across the herpesvirus family and plays a uniformly critical role in silencing viral gene expression for latency (Reeves and Sinclair, 2013; Lieberman, 2015; Cliffe and Wilson, 2017; Hopcraft et al., 2018). In HSV-1 infection, the genome is chromatinized as facultative heterochromatin, which is marked by methylation of lysine 27 on histone 3 (H3K27me3). Facultative chromatin is critical to the regulation of gene expression and is thought to be readily converted to euchromatin to activate gene expression (Trojer and Reinberg, 2007). Therefore, facultative heterochromatin may poise the herpesvirus genomes to readily respond to host cues for reactivation and may contribute to low level or sporadic gene expression during latency (Cliffe et al., 2009, 2013). Heterochromatinization of the whole HCMV genome remains to be fully characterized. Histone deacetylase activity reduces viral gene expression in models for latent HCMV infection (Wright et al., 2005; Saffert et al., 2010), corresponding to increased association of heterochromatin protein 1 (HP1) (Murphy et al., 2002) and the co-repressor KAP1 (Rauwel et al., 2015) with the MIE locus to contribute to silencing the viral genome. KAP1 initiates the formation of heterochromatin by recruiting HP1a and SETDB1 to trigger H3K9 methylation. Further, the polycomb repressive complex 2 (PRC2) increases H3K27me3 marks on the HCMV genome in undifferentiated cell line models of latency (Abraham and Kulesza, 2013). When PRC2 is inhibited, H3K27me3 marks associated with the viral genome decrease and viral gene expression increases, indicative of a role for PRC2 in chromatinization and repression of the viral genome for the establishment of latency.

Many host factors with the potential to suppress viral gene expression are components of PML or ND10 bodies, including PML, Daxx, and Sp100 (Saffert and Kalejta, 2006, 2007; Woodhall et al., 2006). While knockdown of Daxx increases IE gene expression in THP-1 or CD34+ cells infected with the AD169 laboratory strain (Saffert and Kalejta, 2007), it fails to rescue IE expression in cells infected with a low-passage strain (Saffert et al., 2010) or in undifferentiated THP-1 (Wagenknecht et al., 2015) or NTera2 (Groves and Sinclair, 2007) cells. These findings collectively, and not surprisingly, indicate that the repression of gene expression for latency is multifactorial and complex. Despite the ability of the pp71 tegument protein to stimulate viral gene expression by antagonizing Daxx, pp71 fails to traffic to the nucleus and degrade Daxx in cells that support latency (Saffert et al., 2010), a restriction that is overcome by higher multiplicities of infection or virions with high levels of pp71 (Woodhall et al., 2006; Chaturvedi et al., 2020).

Type 1 interferon (IFN) response upregulates PML-associated host factors and reversibly blocks IE gene expression to drive latency in MCMV-infected endothelial cells (Dag et al., 2014). In the context of HCMV infection, US28-mediated downregulation of IFN responsive genes is required for latency and IFI16 stimulates IE gene expression via NFkB for reactivation (Elder et al., 2019). In HSV-1 infection, IFI16 restricts HSV-1 gene expression and is targeted by ICP0 for destruction to stimulate gene expression (Orzalli et al., 2012; Merkl and Knipe, 2019). These studies suggest a complex role for the IFN response in contributing to latency, which is an area of important ongoing research in the field.

SAMHD1 is a restriction factor that depletes the pool of available dNTPs to suppress DNA polymerase processivity and has recently been shown to restrict MIE gene expression and replication by impeding NFkB activation in myeloid cells (Kim et al., 2019). In primary human macrophages, HCMV counteracts this restriction by inducing phosphorylation of SAMHD1 (T592) via the activity of the viral protein kinase, UL97, or activation of CDKs (Businger et al., 2019). HCMV infection also downregulates SAMHD1 transcript and protein levels. This viral strategy is conserved in MCMV where the M97 kinase phosphorylates SAMHD1 (Deutschmann et al., 2019). These findings indicate a role for SAMHD1 in suppressing replication for latency and SAMHD1 inactivation is required for reactivation. Further, the phosphatase CDC25B and CDK1 are repressive to viral gene expression, and inhibition of CDK1 stimulates viral gene expression in the Kasumi-3 model of latency (Pan et al., 2016). These studies collectively illustrate the complex and multi-layered approach to silencing viral gene expression for the establishment of latency.

While much remains to be understood about the signaling events and coordination of repressive activities to repress viral gene expression for latency, much less is known about how these layers of control are unraveled for reactivation. Reactivation necessarily depends on counteracting the strong, layered epigenetic silencing associated with latency. The reactivation of CMV from latency has been intimately linked to changes in signaling and hematopoietic differentiation (Soderberg-Naucler et al., 1997, 2001; Reeves and Compton, 2011; Huang et al., 2012; Kew et al., 2014; Buehler et al., 2016, 2019; Crawford et al., 2018; Forte et al., 2018; Mikell et al., 2019). Differentiation of latently infected cells along the myeloid lineage results in the re-expression of viral genes and reactivation (Ibanez et al., 1991; Taylor-Wiedeman et al., 1994; Soderberg-Naucler et al., 1997, 2001; Reeves and Sinclair, 2013). The chromatin remodeling associated with HCMV reactivation during hematopoietic differentiation is incompletely defined compared to the silencing events that drive latency (Reeves et al., 2005; Dupont et al., 2019). One driver of chromatin remodeling during reactivation is the FACT (facilitates chromatin transcription) complex, which functions to reposition histones and to increase accessibility to RNA polymerase. FACT is bound to the MIE locus both during latency and following reactivation events in Kasumi 3 cells, and is important for the re-expression of IE genes (O'Connor et al., 2016). Other viral proteins, including UL7, UL135 and specific isoforms of UL136, have also been shown to be required for reactivation (Umashankar et al., 2014; Caviness et al., 2016; Crawford et al., 2018; Rak et al., 2018; Mikell et al., 2019). HCMV-coded miRNAs have also emerged as important regulators of host signaling pathways that contribute to reactivation (Mikell et al., 2019).

Reactivation is associated with changes in the levels or binding of host transcriptional activators to the MIE region that stimulates reactivation (Bain et al., 2003; Liu et al., 2008; Kew et al., 2014; O'Connor et al., 2016; Krishna et al., 2019) and contains a high density of binding sites for host transcription factors important in inflammation and differentiation (Collins-McMillen et al., 2018). As an example, the CREB host transcription factor binds to CRE sites within the MIE enhancer and also recruits mitogen and stress activated kinases that initiate chromatin remodeling, resulting in increased IE gene expression in infected monocytes as they differentiate into mature dendritic cells (Kew et al., 2014). Studies in NT2 cells differentiated with phorbol ester treatment have defined a cooperative role for CREB and an additional host transcription factor NF-κB in driving re-expression of the IE genes (Liu et al., 2010; Yuan et al., 2015). However, most, if not all, studies assessing MIE activity following reactivation demonstrate only the accumulation of IE1 and IE2 transcripts or protein, and MIEP activity is presumed without being definitively demonstrated.

Because the MIEP is strongly repressed during latency and chromatin modifications in the MIE region correlate with cellular differentiation and the permissive state, it has been presumed that de-repression of the MIEP is a prerequisite to re-expression of IE genes upon reactivation (Figure 1A). Recent studies identified MIE transcripts encoding IE1-72kDa and IE2-86kDa proteins that have unique 5′ ends compared to those expressed from the MIEP (Arend et al., 2016). Two alternative transcriptional start sites were mapped within intron A of the MIE locus and correspond to two putative promoters, intronic promoters 1 and 2 (iP1 and iP2). The first initiates 350 nucleotides downstream/3′ of the canonical exon 1 splice donor site and has an untranslated region of 378 nucleotides. The second initiates 54 nucleotides upstream/5′ of the canonical exon 2 spice acceptor site and has an untranslated region of 70 nucleotides. Both of these transcripts lack the MIE exon 1. However, as exon 1 is non-coding, these transcripts still support synthesis of full-length IE1 and IE2 proteins. Intriguingly, these transcripts accumulate late in infection following the onset of viral genome synthesis in fibroblasts and their accumulation can be blocked with inhibitors of viral genome synthesis (Arend et al., 2016). The expression of these alternative MIE transcripts correlates with the second phase of IE2 protein accumulation that occurs late in infection of fibroblasts (Arend et al., 2016; Collins-McMillen et al., 2019) and is consistent with continued IE gene expression despite cis repression of the MIEP late in infection (Pizzorno and Hayward, 1990; Cherrington et al., 1991; Liu et al., 1991; Macias and Stinski, 1993; Reeves et al., 2006; Teng et al., 2012). While the promoter sequences present in intron A (herein referred to collectively as intronic promoters) await fine mapping, they are capable of driving IE gene expression outside the context of infection and from constructs containing the entire MIE locus (enhancer to 3′ UTR) where the MIEP core promoter (−94 to +64 relative to the transcription start site) has been deleted (Arend et al., 2016; Hale et al., 2020). These findings suggest that while the MIEP drives robust IE gene expression early in infection, additional promoters drive sustained IE gene expression late in infection and are presumably subject to distinct regulation. It should also be noted that CTCF binds within Intron A (+834 to 852, between iP1 and iP2) to negatively regulate MIE gene expression (Martinez et al., 2014). Disruption of CTCF binding increased MIE gene expression, but conveyed only a modest replicative advantage in fibroblasts and has not been analyzed in other contexts. The deletion of the intronic promoters also disrupts the CTCF binding site within intron A.

**Figure 1:**
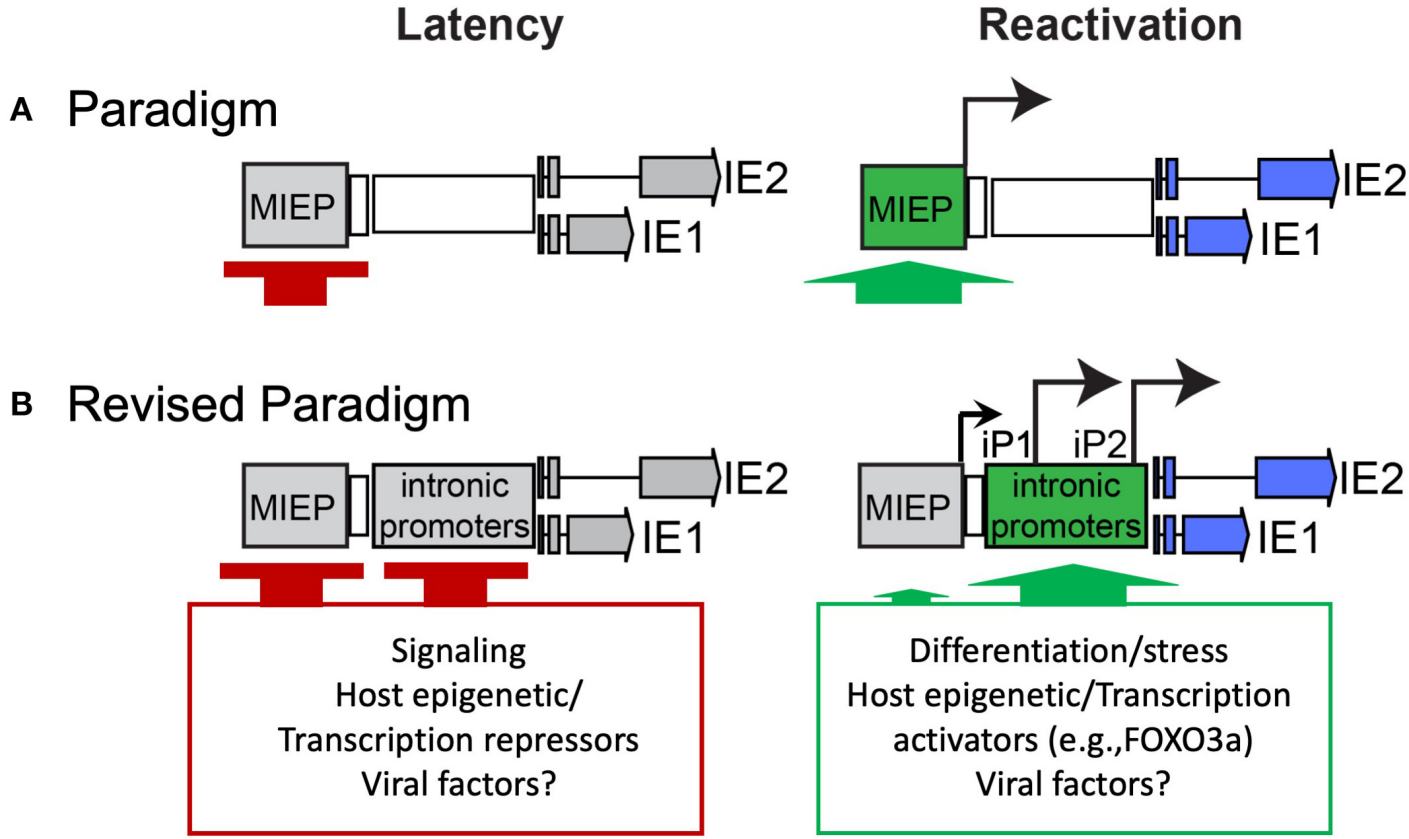
Paradigms of HCMV latency and reactivation. **(A)** Silencing of the MIEP is required for HCMV latency, and it has long been thought that reactivation depends on de-repression of the MIEP for re-expression of IE genes. **(B)** Our recent work sheds new light on the control of MIE gene re-expression in THP-1 cell line and CD34^+^ primary cell models of latency and reactivation. Specifically, it defines alternative promoters within intron A of the MIE locus that give rise to full length IE1 and IE2 proteins. The intronic promoters must also be silenced for latency, similar to the MIEP. An additional post-transcriptional/translational regulation is likely involved, as low levels of iP2-derived transcripts are present during latency. Strikingly, reactivation stimuli in both models induce re-expression of IE1 and IE2 predominantly from the intronic promoters and to a much lesser extent, if at all, from the MIEP. The activation of the intronic promoters is regulated, at least in part, by the host transcription factor, FOXO3a, associated with hematopoietic differentiation. Other viral and cellular factors likely contribute to regulation of the MIE intronic promoters.

While MIE gene expression from the intronic promoters is not required for virus replication in fibroblasts, the presence of these alternative MIE promoters offers exciting possibilities with regard to the control of MIE gene expression in the context of latency and reactivation. This is particularly true in light of findings that MIEP activity is weak in cells that support HCMV latency, including CD34^+^ HPCs and CD14^+^ cells (Shelbourn et al., 1989; Sinclair et al., 1992), which has precluded use of the MIEP to drive transgene expression for gene therapy. Using the THP-1 model for latency, we have shown that a transient burst of IE gene expression occurs immediately following infection but is gradually silenced. Following the stimulation of reactivation with phorbol ester, re-expression of MIE genes is induced. The MIE transcripts expressed in this model originate predominantly from the intronic promoters, not the MIEP (Collins-McMillen et al., 2019). Following reactivation of HCMV in CD34+ HPCs infected *in vitro*, MIE transcripts are also predominantly derived from the intronic promoters, and mutant viruses wherein the intronic promoters are disrupted reactivate poorly following stimulation in infected THP-1 or CD34^+^ HPCs.

These findings suggest a model whereby the MIEP is silenced upon the establishment of latency and re-expression of MIE genes and reactivation of the replicative cycle requires switching to the intronic promoters to drive MIE gene expression (Figure 1B). At least in the contexts we have tested to date, no substantial re-expression from the MIEP has been detected. However, these findings do not preclude the possibility that robust reactivation stimuli lead to re-expression from the MIEP to drive virus replication. In a recent study by Mason and colleagues, transcription from both the MIEP and iP2 was observed in the THP-1 model and in CD14^+^ monocytes treated with phorbol ester (Mason et al., 2020). Further, in dendritic cells derived from either CD34^+^ or CD14^+^ cells and in CD14^+^-derived macrophages stimulated with IL-1b and M-CSF, MIE transcripts were predominantly derived from the MIEP. These findings raise the possibility that regulation of the MIE locus, including the usage of various transcription factor binding sites and distinct promoters, is cell type-specific. This level of complexity would allow the virus to respond to multiple reactivation stimuli using different signaling pathways. In support, the transcription factors CREB and NF-κB, which bind the MIE enhancer region upstream of the MIEP transcription start site, were shown to play a cooperative role in re-initiating MIE gene expression in NT2 cells treated with phorbol ester (Liu et al., 2010; Yuan et al., 2015), whereas deletion of the CREB, but not the NF-κB, binding sites resulted in decreased MIE expression in dendritic cells (Kew et al., 2014). It is also possible that host transcription factor binding sites in the MIE enhancer contribute to changes in 3D chromatin structure and the activity of other regions of the MIE locus, such as the alternative promoters, to drive MIE re-expression. In fact, the activator protein-1 (AP-1) transcription factor binding site in the MIE enhancer has been recently shown to stimulate activation of a MIE intronic promoter, in addition to the MIEP and distal promoter (Krishna et al., 2020). Regardless, our studies indicate an important role for the intronic promoters in re-expression of MIE genes and in reactivation from latency. This promoter switching mechanism provides the versatility necessary to strongly repress the MIE gene expression for latency, while preserving responsiveness to specific host cues for reactivation.

Providing insight into the regulation of the intronic promoters, intron A sequences contain consensus binding sites for the forkhead family transcription factors. The MIE intronic promoters can be activated by both FOXO1 and FOXO3a members; however, FOXO3a appears to be the transcription factor critical for reactivation (Hale et al., 2020). Mutating FOXO binding sites in the MIE intronic promoters diminishes reactivation of HCMV from latency in THP-1 cells or CD34^+^ HPCs, demonstrating a role for FOXO transcription factors in reactivation. This is intriguing, as FOXO3a localizes to the nucleus or is induced in myeloid progenitor cells in response to cellular stresses and differentiation (Paik et al., 2007; Tothova and Gilliland, 2007; Yalcin et al., 2008; Liang et al., 2016). Further, FOXO3a expression is induced by differentiation stimuli in THP-1 cells (Hale et al., 2020). These findings suggest a mechanism by which the virus can sense and respond to host cues for reactivation through FOXO3a activation of the MIE intronic promoters.

Transcripts with alternative 5′ ends may be driven by promiscuous initiation of transcription or alternative promoter usage. Either mechanism serves to provide additional, context-dependent regulation of gene expression or to expand the limited coding potential within a viral genome. The ~240 base pair HCMV genome is estimated to encode >165 ORFs (Davison et al., 2003; Murphy et al., 2003a,b) and as many as 604 non-canonical ORFs (Stern-Ginossar et al., 2012). Over 7,000 transcriptional start sites are detected during lytic replication, a number far exceeding the number of ORFs that have been predicted. RNA polymerase II initiation of HCMV transcription is pervasive (Stern-Ginossar et al., 2012; Parida et al., 2019), with transcriptional start sites clustering within a 20-basepair interval. This indicates that HCMV may have regions to initiate transcription, rather than precise transcriptional start sites (Parida et al., 2019). Some of these start sites may produce alternative 5′ UTRs for differential regulation of translation, affecting ribosome initiation and progression (Mizrahi et al., 2018). Indeed, the 5′ UTRs of MIE transcripts play important roles in regulating the efficiency of translation and enhance translation in the context of infection (Arend et al., 2018).

*UL136* is encoded on a polycistronic locus spanning *UL133-UL138* that encodes multiple proteins important to viral latency and reactivation. UL136 protein is not synthesized from longer transcripts encoding *UL133* or *UL135* (Grainger et al., 2010; Umashankar et al., 2011). Rather, UL136 is synthesized from multiple viral transcripts with unique 5′ ends (Caviness et al., 2014). These transcripts accumulate robustly in the late phase and depend on viral genome synthesis, whereas UL133, UL135, and UL138 are expressed with early kinetics. Unlike the MIE transcripts, which have identical coding sequences resulting in synthesis of either IE1-72kDa or IE2-86kDa whether derived from the MIEP or from the intronic promoters, the nested *UL136* transcripts have unique 5′ ends that give rise to a series of unique proteins, each with a successive truncation amino terminal end compared to the full-length UL136 protein (Caviness et al., 2014). The *UL136* promoter has not been mapped and it is not yet clear if *UL136* transcripts arise from multiple promoters or initiation of transcript or how they might be differentially regulated in different contexts of infection. However, mutant viruses lacking single or combinations of the UL136 variants have revealed important roles for each in either stimulating or suppressing virus replication in endothelial cells and for the establishment of latency or reactivation from latency in CD34+ HPCs and humanized mice (Caviness et al., 2016).

Alternative promoters play key roles in tailoring gene expression for cell lineages, tissue types, developmental stages, stress and differentiation (Davuluri et al., 2008). Although not well characterized for HCMV, other herpesvirus family members also use alternative promoters for context-dependent regulation of gene expression. The IE transactivator, ICP27, of Marek's disease virus is expressed by different promoters in both the lytic and latent contexts of infection (Strassheim et al., 2016). Also, ORF50 of Kaposi's Sarcoma-associated herpesvirus (KSHV) is expressed from a series of transcripts driven by four distinct promoters, giving rise to six differentially spliced ORF50 transcripts, which results in multiple isoforms of the major transactivator RTA (Wakeman et al., 2017). RTA isoforms regulate the KSHV cascade of gene expression, each driving distinct transactivation programs for reactivation, and exhibit variable activity in different cell types. Finally, Epstein-Barr Virus (EBV) uses promoter switching for its distinct transcriptional programs for latency, and specifically the expression of the EBNA-1 latency protein (Woisetschlaeger et al., 1990; Schaefer et al., 1995; Nonkwelo et al., 1996; Tempera et al., 2011). The significance of these promoter switching events to the viral gene expression program, and how they are regulated remains to be fully understood. However, from these limited examples, and now the demonstration of promoter switching events in regulation of MIE gene expression for HCMV reactivation, it appears that promoter switching is a prominent mechanism for regulating gene expression for latency and reactivation.

## Author Contributions

FG wrote manuscript. DC-M and NM contributed data supporting manuscript and revised manuscript. JK contributed data supporting manuscript, revised manuscript, and contributed to figure. All authors contributed to the article and approved the submitted version.

## Conflict of Interest

The authors declare that the research was conducted in the absence of any commercial or financial relationships that could be construed as a potential conflict of interest.
